# Metabolomics-Based Frailty Biomarkers in Older Chinese Adults

**DOI:** 10.3389/fmed.2021.830723

**Published:** 2022-01-26

**Authors:** Yiming Pan, Yun Li, Pan Liu, Yaxin Zhang, Bowen Li, Zuyun Liu, Guanghou Shui, Lina Ma

**Affiliations:** ^1^Department of Geriatrics, National Research Center for Geriatric Medicine, Xuanwu Hospital, Capital Medical University, Beijing, China; ^2^LipidALL Technologies Company Limited, Changzhou, China; ^3^Center for Clinical Big Data and Analytics, School of Public Health, Second Affiliated Hospital and Department of Big Data in Health Science, Zhejiang University School of Medicine, Hangzhou, China; ^4^State Key Laboratory of Molecular Developmental Biology, Institute of Genetics and Developmental Biology, Chinese Academy of Sciences, Beijing, China

**Keywords:** frailty, metabolomics, physical function, biomarker, older adults

## Abstract

**Background/Objectives:**

Owing to accelerated population aging, health in older adults is becoming increasingly important. Frailty can reflect the health status and disease risks of older adults; however, appropriate biomarkers for early screening of frailty have not been identified. Here, we applied metabolomics to identify frailty biomarkers and potential pathogenic mechanisms of frailty.

**Methods:**

Serum metabolic profiles from 25 frail and 49 non-frail (control) older adults were systematically investigated by liquid chromatography-mass spectrometry-based metabolomics.

**Results:**

We identified 349 metabolites of 46 classes, with four increased and seven decreased metabolites in frail older adults. Pearson correlation analysis identified 11 and 21 metabolites that were positively and negatively correlated with grip strength, and 7 and 76 metabolites that were positively and negatively correlated with gait speed, respectively. Pathway analysis identified 10 metabolite sets and 13 pathways significantly associated with one or more frailty phenotype criteria.

**Conclusion:**

These results revealed the metabolite characteristics of serum in frail older adults. Intermediates of carbohydrate metabolism (e.g., isocitrate, malate, fumarate, cis-aconitate, glucuronate, and pyruvate), saturated fatty acids (e.g., palmitic acid), unsaturated fatty acids (e.g., arachidonate and linoleic acid), and certain essential amino acids (e.g., tryptophan) may be candidate biomarkers for the early diagnosis of frailty. Mitochondrial function disorders, saturated fatty acid-mediated lipotoxicity, aberrant unsaturated fatty acid metabolism, and increased tryptophan degradation could be potential mechanisms of frailty.

## Introduction

Aging is an inevitable life process, characterized by a time-dependent decline in functional capacity and stress resistance associated with increased risks of morbidity and mortality (1). Preventing aging-related damage, disease, and disability in older adults has become a priority in the public health sector. However, the functional decline of an individual is only loosely consistent with the advancement in chronological age (2). Frailty can better reflect the physiological status and disease risk of older adults than the chronological age (3). Therefore, the early screening of frailty is critical for personalized intervention in age-related diseases and the prevention of adverse outcomes. Given the lack of a unified definition for frailty, researchers commonly identify frailty using different assessment tools, such as the Fried frailty phenotype (4) and the frailty index (5). However, these assessment tools are not objective enough. Sensitive and specific biomarkers for frailty are urgently needed for more accurate identification in older adults (6).

Currently, studies on frailty biomarkers suggest that frailty involves crosstalk between multiple physiological pathways and various molecular changes (7). Instead of a single biomarker, a group of biomarkers may be more promising for the identification of frailty (8). However, no frailty biomarkers have been widely recognized, and the relationships between candidate biomarkers and frailty phenotypes are largely unknown. These issues represent major challenges in the field.

Metabolomics is a platform used to analyze the terminal metabolites of different samples from diverse organisms. This approach has promising applications in the elucidation of the metabolic spectrum among older adults and could facilitate the identification of the pathways underlying frailty (9). Recent metabolomics studies have demonstrated strong associations between certain metabolites and frailty. However, most of these studies were performed in European and American populations, and most of them only apply targeted metabolomics analyses for some metabolites, leading to a lack of clear consensus among studies (10–14).

Thus, we hypothesized that frailty may involve characteristic metabolites, which may contribute to the early identification and personalized intervention of frailty. Accordingly, in the current study, we used untargeted metabolomics platforms to analyze metabolites in the serum of frail and non-frail Chinese older adults to identify potential biomarkers of frailty. We expect that our findings may provide insights into the underlying biological pathways involved in frailty and identify effective targets for the treatment of age-related diseases.

## Materials and Methods

### Participants

In total, 74 participants aged 60 years and older were recruited for this study. The mean age was 76.34 ± 8.31 years, and 64.9% of the participants were men. Individuals with cancer, rheumatic diseases, severe infections, severe liver (with Child-Pugh class B or C) or renal (with GFR <60 mL/min/1.73 m^2^) insufficiency, or receiving hormone or immunosuppressive therapy were excluded. All participants were divided into two groups: the frail group (case group, *n* = 25) and the non-frail group (control group, *n* = 49). Age and sex were matched between the two groups. All subjects gave written informed consent following the Declaration of Helsinki and the study was approved by the ethical review board of Xuanwu Hospital Capital Medical University with the approval number of [2020]043.

We collected data on general information (including age, sex, education, height, body weight, smoking, and drinking), blood pressure, medical history of chronic diseases, and some laboratory test results from each participant. The body mass index (BMI) was calculated as the weight in kilograms divided by the square of the height in meters.

### Frailty Assessment

Frailty status was assessed according to the frailty phenotype (4) composed of five criteria: weakness, slowness, inactivity, fatigue, and shrink. The standing grip strength of both hands was measured twice, and the maximum value was adopted. Weakness was defined after adjusting for sex and BMI (15). Gait speed was measured with a 4-m-walk test at the usual speed. The cutoff point of slowness was adjusted for sex and height (15). Inactivity was defined as not walking 2.0 h per week. Fatigue was identified if participants said, “I felt everything I did was an effort” or “I could not get going” more than 3 days per week. Shrink was defined as the unintentional loss of at least 5% of weight from the previous year or BMI <18.5 kg/m^2^. Participants with poor performance in three or more criteria were defined as frail, and those with two or fewer were defined as non-frail (4).

### Serum Sample Collection

All blood samples were collected using serum separation tubes between 6:00 and 6:30 a.m. after overnight fasting, and the serum was extracted and stored in centrifuge tubes at −80°C.

### Metabolomics Analysis

#### Reagents

Water was purified using an ultrapure water preparation system. Liquid chromatography-mass spectrometry (LC-MS) grade acetonitrile and methanol were purchased from Merck (Germany). High-performance LC-grade formic acid was obtained from Sigma (Germany). All internal standard references were purchased from Cambridge Isotope Laboratories (USA).

#### Metabolome Analysis

The extraction protocol and metabolomics are described as previously reported (16). Briefly, 50 μl serum was added to 200 μl ice-cold methanol, incubated for 30 min at 1,500 rpm and 4°C, and centrifuged for 10 min at 12,000 rpm and 4°C. The supernatant was removed into a clean 1.5-ml centrifuge tube and dried using a SpeedVac (Genevac miVac, Tegent Scientific Ltd., England), and the dried extracts were redissolved in 1% acetonitrile in water. The upper layer liquids were collected for LC-MS analysis. Quality control (QC) samples were prepared by mixing all serum samples in steps identical to those for the actual serum samples. In order to judge the stability of the instrument, one QC sample was tested after every 10 samples, and there was also one QC sample tested before the first and after the last sample, respectively. ACQUITY UPLC HSS T3 (1.8 μm, 2.1 × 100 mm) columns (Waters, Dublin, Ireland) were used in the current study. Ultra Performance Liquid Chromatography (UPLC) (Agilent 1290 Infinity II; Agilent Technologies, Germany) coupled with high-resolution mass spectrometry (5600 Triple TOF Plus, AB Sciex, Singapore) was used to acquire the metabolome data. The temperatures of the column and auto-sampler were maintained at 40 and 4°C, respectively. The injection volume was 5 μL per run, and the flow rate was 0.35 mL/min. The MS parameters for detection were: ESI (-) source voltage −4.5 kV and +5.5 kV for ESI (+); vaporizer temperature, 500°C; drying gas (N2) pressure, 50 psi; nebulizer gas (N2) pressure, 50 psi; curtain gas (N2) pressure, 35 psi; the scan range was m/z 60–600. Information-dependent acquisition mode was used for MS/MS analyses of the metabolites. The collision energy was set at 35 ± 15 eV. Data acquisition and processing were performed using Analyst TF 1.7.1 (AB Sciex, Concord, ON, Canada). Each sample was tested once.

Metabolite identification was compared with standard references, HMDB (the Human Metabolome Database) and METLIN (the METLIN Metabolite and Chemical Entity Database). A total of 48 isotopically labeled internal standards were spiked into the samples for the semi-quantification of metabolites. Metabolite intensities were normalized according to the following rules and referred to as intensity (16).

### Statistical Analysis

For basic information, continuous variables were presented as means and SD, and categorical variables were presented as numbers and percentages (%). The student's *t*-test was used to compare the continuous variables, and Pearson's χ^2^ test was used to compare the categorical variables. Pairwise comparison was used to test the consistency of QC and the stability of the instrument. For metabolomics datasets, missing values were replaced with 0. Sparse partial least squares (sPLS) regression was used to observe the differentiation of samples within groups, and the association between differential metabolites and frailty phenotypes with R package mixOmics Version 6.16.3 (17). We used the *limma* package Version 3.48.3 of R (18) to identify differentially expressed metabolites between frail and non-frail groups, as well as participants with each frailty criterion and their counterparts without that criterion, respectively. We made Logistic regression on the different metabolites identified and receiver operating characteristic (ROC) analysis on the diagnostic models. Pearson correlation analysis was used to identify correlations between metabolites and physical functions (gait speed and grip strength). Differences were considered to be statistically significant for a two-tailed *P* < 0.05. All statistical analyses were performed in SPSS Statistics 26 (Armonk, NY: IBM Corp) and R 4.10.

### Pathway Analysis

To identify the metabolic pathways significantly associated with the frailty phenotype, metabolite set enrichment analysis (19) and metabolic pathway analysis (20) were performed based on the Small Molecule Pathway Database (https://smpdb.ca) and the Kyoto Encyclopedia of Genes and Genomes (https://www.kegg.jp/), respectively. Metabolites with a *P* < 0.05 in two-tailed Mann-Whitney U tests were used for pathway analysis, and those with no matched HMDB ID were removed. A pathway with a fold change >2 and a raw *P* < 0.05 in metabolite set enrichment analysis or with a raw *P* < 0.05 in metabolic pathway analysis indicated that it contained more differentially expressed metabolites with respect to the frailty phenotype.

## Results

**Figure 1:**
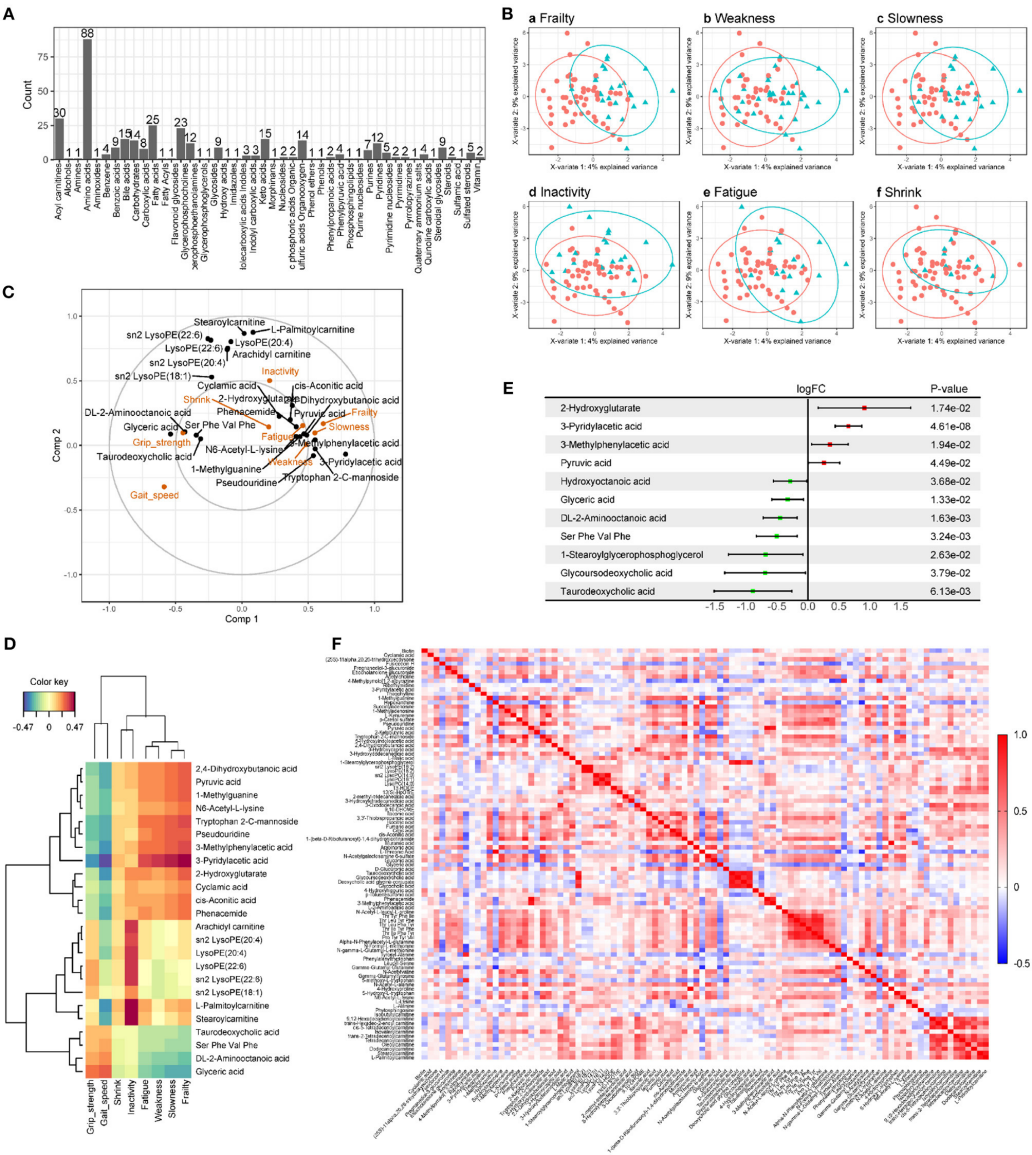
Metabolomics analysis of serum from older adults. **(A)** Metabolome summary. There were 349 metabolites in 46 categories detected from serum samples of participants, among which Amino acids had the most identified species (88). **(B)** The sparse partial least squares (sPLS) regression score plots of the first two components within each pair of groups. The green triangle represents the case group (frailty, weakness, slowness, inactivity, fatigue, and shrink are shown in **Ba–f**), the red circle represents the control group, and the ellipses represent the 95% confidence regions for each group. As the score plots show, the metabolites detected have well-separated within groups. **(C)** Correlation circle plot for the first two sPLS components. The projection of each variable on the axis represents the correlation between the variable and the corresponding component. To simplify the plot, 16 and 8 metabolites were retained in Comp1 and Comp2, respectively. **(D)** Heat map of correlation between the clinical variables associated with frailty phenotype and 24 metabolites from the first two components in sPLS regression. Negative and positive correlations are shown in blue and red ranging from −0.47 to 0.47. **(E)** Forest plot of different metabolites for frailty identified by *limma* package of R with *P* < 0.05. Serum of frail older adults had higher levels of 4 metabolites and lower levels of 7. **(F)** Heat map of Pearson correlation analysis of 96 metabolites associated with gait speed or grip strength. Negative and positive correlations are shown in blue and red ranging from −0.5 to 1.0.

There were no significant differences in age, sex, BMI, smoking, drinking, blood pressure, chronic diseases, and laboratory tests between the frail and non-frail groups (Table 1). A pairwise comparison of QC showed good consistency and high data quality (Supplementary Figure S1). UPLC-quadrupole time-of-flight (QTOF) mass spectrometry-based untargeted metabolomics platform analysis identified 349 metabolites of 46 categories (Figure 1A). Sparse partial least squares (sPLS) regression shows that the subjects can be moderately well-separated based on frailty status, as well as by their individual indices, including weakness, slowness, inactivity, fatigue, and shrink (Figure 1B). As the correlation circle plot for the first two sPLS components shown in Figure 1C, frailty had a positive correlation with each frailty phenotype criteria and a negative correlation with gait speed and grip strength. Besides, Figure 1C showed associations between frailty phenotypes and metabolites. Metabolites including pyruvic acid, dihydroxybutanoic acid, 1-methylguanine, etc. showed a positive correlation with frailty, slowness, weakness, and fatigue, while glyceric acid, DL-2-aminooctanoic acid, etc., showed a positive correlation with grip strength. A group of lysophospholipids and acyl-carnitine were clustered along with the 2nd component which indicated a positive correlation with inactivity and a negative correlation with gait speed. The heat map showed the correlation between frailty phenotype and 24 metabolites from the first two components in sPLS regression in color (Figure 1D). Compared with non-frail participants, frail older adults showed significant suppression and enhancement of seven and four metabolites, respectively (Figure 1E). Moreover, we regrouped participants according to each frailty phenotype domain criteria. There were 19, 18, 46, 6, and 8 different metabolites observed in the weakness, slowness, inactivity, fatigue, and shrink groups, respectively, compared with those in the control groups. The details of the different metabolites are shown in Supplementary Figure S2. We successfully built Logistic regression models of frailty, slowness, weakness, inactivity, and shrink with their differential metabolites, respectively. Their diagnostic efficacy and ROC analysis were shown in Supplementary Figure S3. Pearson's correlation analysis showed that 11 and 21 metabolites were positively and negatively correlated with grip strength, respectively, whereas 7 and 76 metabolites were positively and negatively correlated with gait speed, respectively (Figure 1F, Supplementary Tables S1, S2).

**Table 1:** Comparison of characteristics between non-frail and frail groups.

**Variables**		**Non-frail (*n* = 49)**	**Frail (*n* = 25)**	***P value***
General information	Age (years), mean (SD)	76.22 (8.26)	76.56 (8.58)	0.871
	Male, no (%)	31 (63.3)	17 (68.0)	0.687
	High school and above, no (%)	29 (70.7)	11 (47.8)	0.069
	Body mass index (kg/m^2^), mean (SD)	24.88 (3.80)	25.40 (3.77)	0.580
	Smoking, no (%)	17 (34.7)	11 (44.0)	0.435
	Drinking, no (%)	13 (26.5)	10 (40.0)	0.236
Frailty assessment	Walking speed (m/s), mean (SD)	0.97 (0.25)	0.67 (0.30)	<0.001^*^
	Grip strength (kg), mean (SD)	28.18 (7.94)	21.71 (8.50)	0.004^*^
	Shrink, no (%)	3 (6.1)	6 (24.0)	0.026^*^
	Inactivity, no (%)	6 (12.2)	15 (60.0)	<0.001^*^
	Self-reported fatigue, no (%)	24 (49.0)	17 (68.0)	0.119
Blood pressure	Systolic blood pressure (mmHg), mean (SD)	139 (16.04)	141 (24.00)	0.751
	Diastolic blood pressure (mmHg), mean (SD)	74 (11.20)	75 (11.46)	0.757
Chronic diseases	Hypertension, no (%)	38 (77.6)	21 (84.0)	0.514
	Diabetes mellitus, no (%)	17 (34.7)	9 (36.0)	0.911
	Coronary heart disease, no (%)	19 (38.8)	13 (52.0)	0.277
	Pulmonary disease, no (%)	7 (14.3)	2 (8.0)	0.434
	Chronic kidney disease, no (%)	3 (6.1)	1 (4.0)	0.703
Laboratory tests	Cholesterol (mmol/L), mean (SD)	3.97 (0.82)	4.16 (1.07)	0.424
	Triglycerides (mmol/L), mean (SD)	1.38 (0.92)	1.66 (1.21)	0.277
	Low density lipoprotein (mmol/L), mean (SD)	2.32 (0.70)	2.56 (0.94)	0.235
	High density lipoprotein (mmol/L), mean (SD)	1.24 (0.35)	1.16 (0.31)	0.373
	Hemoglobin A1c (%), mean (SD)	6.26 (1.27)	6.65 (1.65)	0.265
	Fasting plasma glucose (mmol/L), mean (SD)	5.62 (1.86)	6.43 (3.02)	0.228

* *p < 0.05*.

Next, we generated metabolite sets for enrichment analysis and metabolic pathway analysis. Table 2 shows metabolite sets with a fold change of >2 and a raw *P* < 0.05 from the enrichment analysis for each frailty phenotype. The two most significantly enriched metabolite sets were fatty acid metabolism (upregulation of l-carnitine, palmitic acid [PA], and l-palmitoylcarnitine) and mitochondrial beta-oxidation of long-chain saturated fatty acids (upregulation of l-carnitine and stearoylcarnitine). Table 3 presents 13 pathways that involved at least one metabolite associated with one or more frailty phenotype criteria. The top three significant pathways were ascorbate and aldarate metabolism, pentose and glucuronate interconversions, and lysine degradation. However, certain pathways associated with the frailty phenotype had low impacts, such as inositol phosphate metabolism, pentose phosphate pathway, vitamin B6 metabolism, fatty acid degradation, and unsaturated fatty acid biosynthesis.

**Table 2:** Enriched metabolite sets in frailty phenotype.

**Frailty phenotype**	**Metabolite set**	**Match status**	**Fold change**	**Metabolites**		**Raw *p***
				**Up-regulated**	**Down-regulated**	
Frailty	Glycolysis	1/1	56.5	Pyruvic acid	–	0.0177
	Pyruvaldehyde degradation	1/1	56.5	Pyruvic acid	–	0.0177
	Glycine and serine metabolism	2/16	7.07	Pyruvic acid	Glyceric acid	0.0190
	Glycerolipid metabolism	1/2	28.25	–	Glyceric acid	0.0352
Slowness	Inositol metabolism	1/1	56.5	D-Glucuronic acid	–	0.0177
	Starch and sucrose metabolism	1/1	56.5	D-Glucuronic acid	–	0.0177
	Glycerolipid metabolism	1/2	28.25	–	Glyceric acid	0.0352
Inactivity	Fatty acid metabolism	3/3	10.27	L-Carnitine, Palmitic acid, L-Palmitoylcarnitine	–	0.0007
	Mitochondrial beta-oxidation of long chain saturated fatty acids	2/2	10.26	L-Carnitine, Stearoylcarnitine	–	0.0087
Fatigue	Vitamin B6 metabolism	1/1	37.74	–	4-Pyridoxic acid	0.0265
Shrink	Transfer of acetyl groups into mitochondria	2/5	7.555	Citric acid	Oxalacetic acid	0.0220

**Table 3:** Over-represented pathways associated with frailty phenotype with raw *P* < 0.05.

**Frailty phenotype**	**KEGG ID**	**Pathway**	**Match status**	**Metabolites**		**Raw *p***	**Impacts score**
				**Up-regulated**	**Down-regulated**		
Frailty	hsa00260	Glycine, serine and threonine metabolism	2/11	Pyruvate (C00022)	D-Glycerate (C00258)	0.0101	0.0242
	hsa00630	Glyoxylate and dicarboxylate metabolism	2/12	Pyruvate (C00022)	D-Glycerate (C00258)	0.0121	0.0794
	hsa00561	Glycerolipid metabolism	1/1	–	D-Glycerate (C00258)	0.0190	0.0935
	hsa00010	Glycolysis / Gluconeogenesis	1/2	Pyruvate (C00022)	–	0.0379	0.1004
	hsa00030	Pentose phosphate pathway	1/2	–	D-Glycerate (C00258)	0.0379	0.0000
Weakness	hsa00310	Lysine degradation	2/4	N6,N6,N6-Trimethyl-L-lysine (C03793), 2-Oxoadipate (C00322)	–	0.0156	0.1409
	hsa00380	Tryptophan metabolism	2/6	5-Hydroxyindoleacetate (C05635), 2-Oxoadipate (C00322)	–	0.0371	0.0139
Slowness	hsa00040	Pentose and glucuronate interconversions	2/2	Beta-D-Glucuronoside (C03033), D-Glucuronate (C00191)	–	0.0011	0.2656
	hsa00053	Ascorbate and aldarate metabolism	1/1	D-Glucuronate (C00191)	–	0.0381	0.5000
	hsa00561	Glycerolipid metabolism	1/1	–	D-Glycerate (C00258)	0.0381	0.0935
	hsa00562	Inositol phosphate metabolism	1/1	D-Glucuronate (C00191)	–	0.0381	0.0000
Inactivity	hsa01040	Biosynthesis of unsaturated fatty acids	3/6	Hexadecanoic acid (C00249), (9Z)-Octadecenoic acid (C00712), Arachidonate (C00219)	–	0.0053	0.0000
	hsa00071	Fatty acid degradation	2/3	Hexadecanoic acid (C00249), L-Palmitoylcarnitine (C02990)	–	0.0148	0.0000
Fatigue	hsa00260	Glycine, serine and threonine metabolism	2/11	–	Betaine (C00719), D-Glycerate (C00258)	0.0285	0.0745
	hsa00561	Glycerolipid metabolism	1/1	–	D-Glycerate (C00258)	0.0286	0.0935
	hsa00750	Vitamin B6 metabolism	1/1	–	4-Pyridoxate (C00847)	0.0286	0.0000

## Discussion

**Figure 2:**
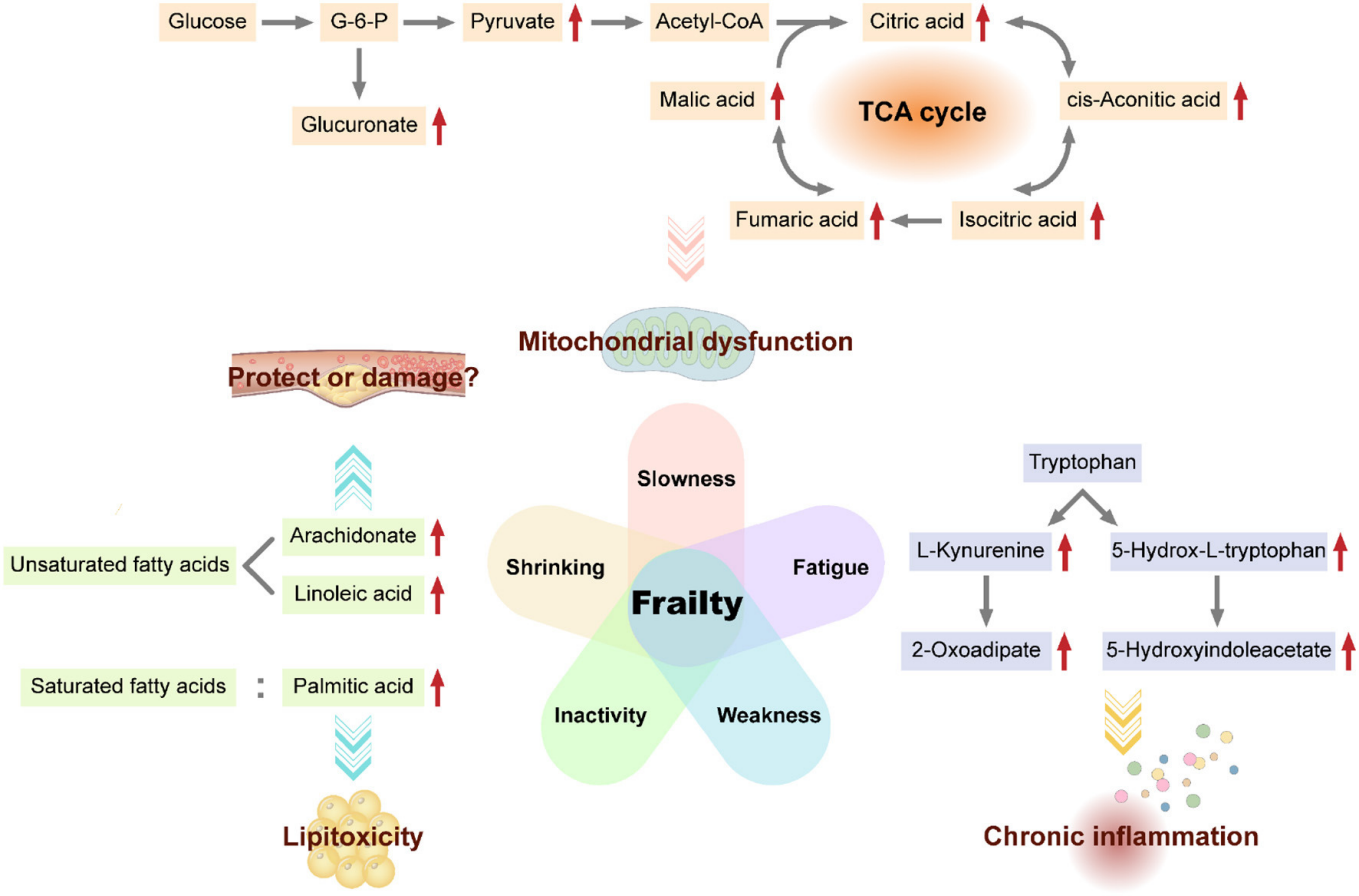
Potential biomarkers and mechanisms of frailty. Some metabolites of carbohydrate metabolism (e.g., glucuronate, pyruvate, citric acid, cis-aconitate, isocitrate, fumarate, and malate), fatty acids (e.g., arachidonate, linoleic acid, and palmitic acid), and certain amino acids (e.g., tryptophan) are candidate biomarkers for frailty. Mitochondrial dysfunction, saturated fatty acid lipotoxicity, cardiovascular effects of unsaturated fatty acids, and chronic inflammation caused by increased tryptophan degradation may be possible mechanisms for frailty.

In this exploratory metabolomics investigation, we showed that frail and non-frail individuals exhibited distinct metabolite signatures. Carbohydrate, lipid, and amino acid metabolism pathways were significantly associated with the frailty phenotype, suggesting that regulation of these pathways may affect frailty (Figure 2).

Carbohydrate metabolism is the main energy source for the body and can provide necessary intermediate metabolites for various important biochemical reactions *in vivo*. The citrate cycle is the ultimate aerobic metabolic pathway of carbohydrate, lipid, and amino acid in the mitochondrial matrix (21). Our results showed a negative correlation between gait speed and isocitrate, l-malic acid, fumarate, and cis-aconitate. Accumulation of these citrate cycle-related metabolites in the serum of frail older adults may result from impaired mitochondrial function and downregulation of the citrate cycle in frailty. Indeed, in the Framingham Offspring Study, researchers found that participants with higher concentrations of isocitrate tended to have lower odds of longevity (beyond the age of 80 years) (22). Additionally, aconitate, isocitrate, and malate were significantly associated with the incidence of all-cause mortality after adjusting for baseline data (22). A study on community-dwelling older black men from America also showed that isocitrate positively correlated with the Scale of Aging Vigor in Epidemiology (SAVE) scores for frailty (23). In addition, Ubaida-Mohien et al. (24) quantitatively characterized the proteins in the citrate cycle and observed that malate dehydrogenase, isocitrate dehydrogenase, fumarate hydratase, and succinate-CoA ligase were significantly lower in older individuals, which indicated decreased mitochondrial function and downregulation of citrate cycle during aging. In addition, we observed that certain carbohydrate metabolism-related pathways, such as glycolysis, gluconeogenesis, pyruvaldehyde degradation, inositol metabolism, ascorbate, and aldarate metabolism, pentose and glucuronate interconversions, and starch and sucrose metabolism, were associated with the frailty phenotype, particularly slowness. The accumulation of D-glucuronate, pyruvate and beta-D-glucuronoside in these pathways may result from impaired mitochondrial function in frailty. Garvey et al. (25) performed a semiquantitative global muscle metabolomics profiling of FBN F1 hybrid male rats and showed that glycolysis intermediates, such as pyruvate, accumulated in the gastrocnemius of aged rats, consistent with mitochondrial dysfunction. In a proteomics analysis of the muscles of a Sod1^−/−^ mouse model of accelerated sarcopenia, enzymes participating in carbohydrate metabolism were downregulated in the case group (26), which may disrupt normal mitochondrial function and result in muscle mass loss. Taken together, these findings suggest that intermediate metabolites of carbohydrate metabolism, such as isocitrate, malate, fumarate, cis-aconitate, pyruvate, and glucuronate, may be potential biomarkers for frailty.

Fat tissue is crucial for energy storage, immune and endocrine processes, thermoregulation, mechanical protection, and tissue regeneration. Fat metabolism disorders with aging lead to fat tissue redistribution in different fat depots, even in non-adipose tissue (27). We observed fat metabolism upregulation in the inactivity group, including both saturated and unsaturated fatty acid metabolism.

In our study, PA levels were higher in the serum of older adults with low physical activity. PA, a common saturated fat, exhibits lipotoxicity, which can induce ectopic lipid deposition and cellular dysfunction (28). PA causes lipotoxicity in tissues, cells, and organs, such as the bone (29), hepatocytes (30), and testes (31), and may increase the risk of diabetes (32). Thus, PA may reflect abnormal fat distribution in frailty. Additionally, rapamycin blocks PA-dependent lipotoxicity in the bones by modulating apoptosis and autophagy through the mammalian target of rapamycin (mTOR) complex 1 pathway (33) as mTOR signaling regulates *de novo* lipid synthesis (21). Further exploration of these relevant pathways may facilitate elucidation of the mechanisms of lipotoxicity and improve our ability to treat or prevent frailty.

Furthermore, we observed higher levels of circulating unsaturated fatty acids, such as arachidonate (AA) and linoleic acid (LA), in frail older adults. AA and LA are common omega-6 polyunsaturated fatty acids (n-6 PUFAs). Our results indicated that higher levels of circulating n-6 PUFAs were characteristic of frailty. The effects of n-6 PUFAs on health have long been controversial. In the Framingham heart study, higher n-6 PUFA intake increased fasting triglyceride levels, remnant-like particle concentrations, and very-low-density lipoprotein sizes. Moreover, n-6 PUFAs decreased low-density lipoprotein size, which may increase the risk of cardiovascular disease (34). Because unsaturated fatty acids are closely related to the differentiation and inflammatory responses of T cells (35), B cells (36), and macrophages (37), the negative effects of n-6 PUFAs on the cardiovascular system may result from increased inflammation activated by it (38). However, other researchers have recently demonstrated the health-protective effects of n-6 PUFAs. For example, higher n-6 PUFA intake could reduce total cholesterol levels in the serum and benefit people at high risk of myocardial infarction (39), could modestly reduce risk of mortality from all causes (40) and have long-term preventive effects on type two diabetes in the population. Higher levels of circulating and tissue LA were associated with a lower risk of major cardiovascular events (41). Overall, although we observed higher levels of circulating unsaturated fatty acids (particularly n-6 PUFAs) in older adults with low physical activity, the clinical significance of these molecules in frail individuals is still unclear. Further research on the related mechanisms is required.

Tryptophan (Trp) is an essential amino acid linked to muscle metabolism (42) and the nervous system (43). We observed upregulation of Trp metabolites, such as 5-hydroxyindoleacetate and 2-oxoadipate, in the serum of weak older adults, although we detected no differences in Trp contents. Trp metabolism is associated with grip strength. Similar to our results, researchers observed that circulating Trp level reduced in frail old patients with breast cancer (44) and frail older black men living in community (23). Even in muscle biopsies, Trp levels were found to be lower in frail older adults (12). The reduction in serum Trp with aging in rats was similar to that observed in humans (25). These studies reported reduction in Trp contents with aging or aggravated frailty, although the mechanisms of this phenomenon are unclear. Based on metabolomics profiling of mice and humans, Westbrook et al. revealed that the tryptophan degradation pathway was significantly activated in frail individuals with down-regulated Trp levels and up-regulated Trp metabolites like kynurenine, which had links with chronic inflammation (45). In our study, Trp metabolite contents increased in the weak group; these results may be explained by enhanced Trp degradation, consistent with the Trp concentration reduction in the aforementioned studies. However, the mechanisms are still not known, and it is unclear whether Trp synthesis is disrupted in frail individuals.

In this study, we explored the metabolic profiles of frailty in older Chinese adults. Our study identified candidate biomarkers for physical frailty, which are of practical value for both clinical diagnosis and basic research on frailty. However, this study has certain limitations. First, the sample size was limited; thus, we did not perform an additional sex control analysis. Second, since this was a single-center study, we did not evaluate people of different regions, races, lifestyles, dietary habits, cultures, and economic levels. Therefore, our results may not represent the entire Chinese population. Despite these limitations, we observed differences between the metabolite profiles in frail and non-frail groups, suggesting that it may be feasible to identify frailty biomarkers through metabolomics platforms. Further studies are needed to test our findings in larger cohorts and more populations, focus on dynamic changes in distinct metabolites and build a diagnostic model of frailty to confirm a set of sensitive and specific biomarkers for early diagnosis. In addition, technological advancements in metabolomics are expected to promote the study of frailty biomarkers.

## Conclusion

Our findings emphasized the value of metabolomics in the search for frailty biomarkers and initially revealed the metabolomic signatures in the serum of frail older adults from China. Isocitrate, malate, fumarate, cis-aconitate, glucuronate, pyruvate, PA, AA, LA, and Trp could be potential candidate biomarkers for frailty. Disorders of mitochondrial function, lipotoxicity of saturated fatty acids, disturbances in unsaturated fatty acid metabolism, and increased Trp degradation were identified as potential mechanisms and therapeutic targets of frailty. Future studies are needed to replicate our results in different populations and provide more evidence on the underlying mechanisms of frailty.

## Data Availability Statement

The raw data supporting the conclusions of this article will be made available by the authors, without undue reservation.

## Ethics Statement

The studies involving human participants were reviewed and approved by the Ethical Review Board of Xuanwu Hospital Capital Medical University. The patients/participants provided their written informed consent to participate in this study.

## Author Contributions

LM: study concept and design. YL: leading of study. YP, PL, and YZ: acquisition of subjects and/or data. YP, BL, ZL, GS, and LM: analysis and interpretation of data, and preparation of the manuscript. All authors critically revised the manuscript for important intellectual content and read and approved the final manuscript.

## Funding

This work was supported by the National Natural Science Foundation of Beijing (7202059, LM), National Key R&D Program of China (2018YFC1312001, LM), Beijing Municipal Hospital Scientific Research Training Project (PX2020036, LM), and Milstein Medical Asian American Partnership Foundation Project Award in Geriatrics (2018, LM; 2020, ZL).

## Conflict of Interest

BL is employed by the LipidALL Technologies Company Limited. The remaining authors declare that the research was conducted in the absence of any commercial or financial relationships that could be construed as a potential conflict of interest.

## Publisher's Note

All claims expressed in this article are solely those of the authors and do not necessarily represent those of their affiliated organizations, or those of the publisher, the editors and the reviewers. Any product that may be evaluated in this article, or claim that may be made by its manufacturer, is not guaranteed or endorsed by the publisher.

## Supplementary Material

Supplementary Figure S1 Pairwise comparison of 13 quality control (QC) samples transformed by log10. Each point in the scatter plot represents a metabolite. All the points are distributed in a tight straight line, and the corresponding correlation coefficient is >0.99, indicating that the data is highly consistent in the two QC samples. On the diagonal is the data distribution diagram of a single sample.

Supplementary Figure S2 Forest plot of different metabolites for each frailty phenotype criteria (weakness, slowness, inactivity, fatigue and shrink) identified by limma package of R with *P* < 0.05. The upregulated metabolites in case groups are shown in red dots and downregulated ones in green.

Supplementary Figure S3 The receiver operating characteristic (ROC) curve of frailty and some frailty phenotype. The figures show the performance of Logistic regression models of differential metabolites in distinguishing frailty, slowness, weakness, inactivity, and shrink from corresponding control groups. The sensitivity, specificity, and area under the ROC curve (AUC) are shown in the images.

Supplementary Table S1 Pearson correlation of metabolites associated with grip strength.

Supplementary Table S2 Pearson correlation of metabolites associated with gait speed.
