# Supplementary figures and images for: Metabolomics-Based Frailty Biomarkers in Older Chinese Adults
Source: Front Med (Lausanne). 2022 Jan 26;8:830723. doi: 10.3389/fmed.2021.830723 (PMC8825494; doi:10.3389/fmed.2021.830723)

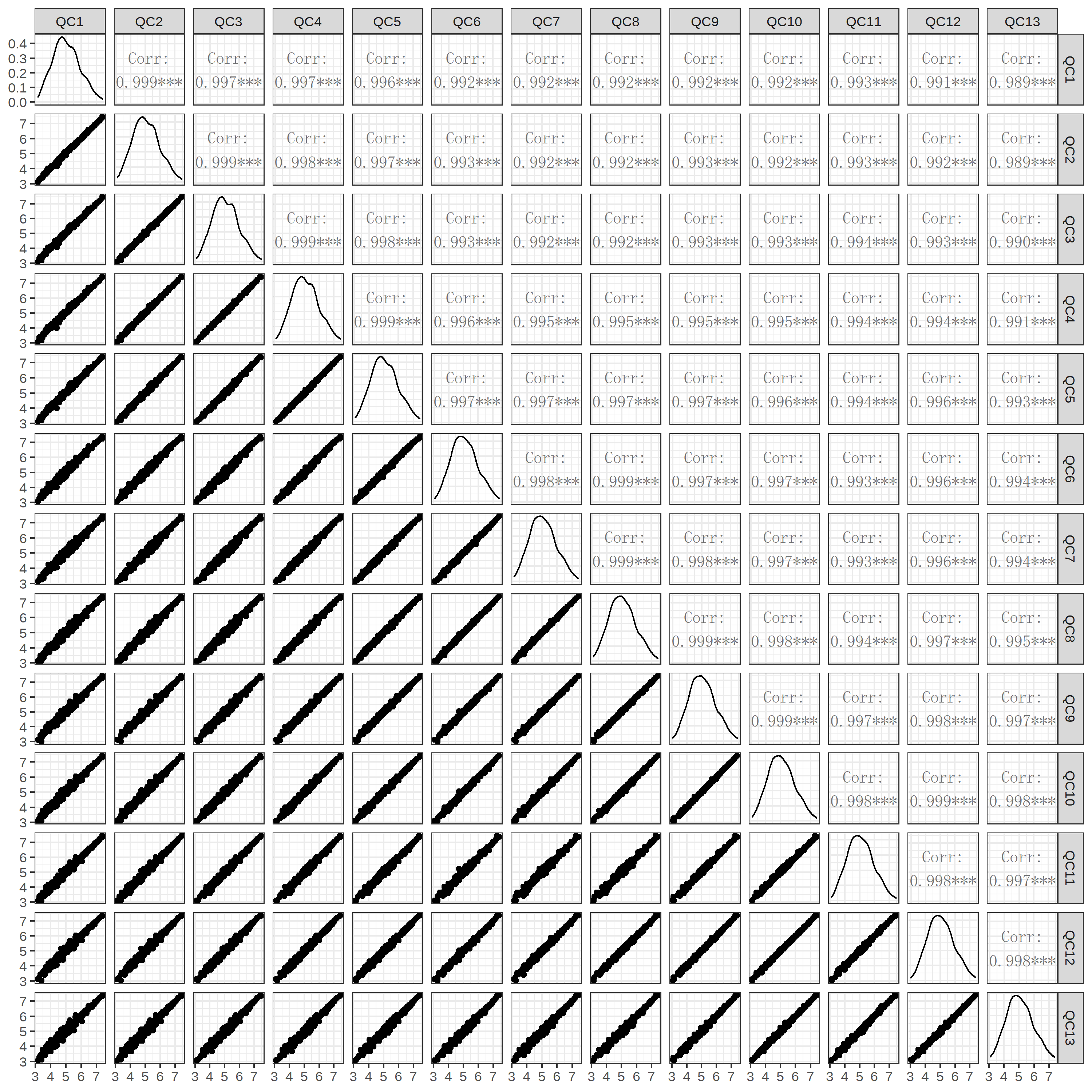

Supplement: Supplementary Figure S1 — Pairwise comparison of 13 quality control (QC) samples transformed by log10. Each point in the scatter plot represents a metabolite. All the points are distributed in a tight straight line, and the corresponding correlation coefficient is >0.99, indicating that the data is highly consistent in the two QC samples. On the diagonal is the data distribution diagram of a single sample. [file Data_Sheet_1.zip › Figure 1.TIFF]

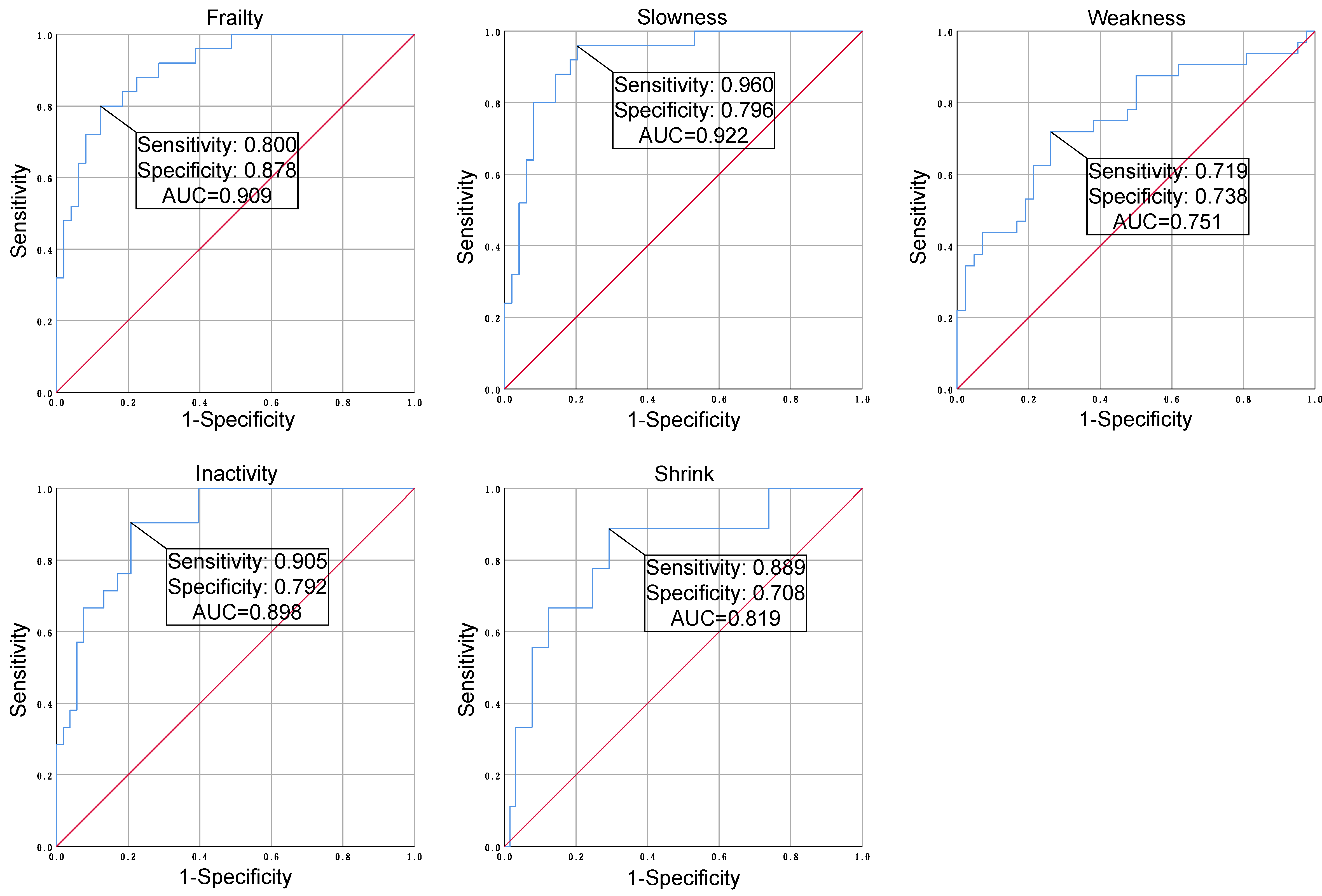

Supplement: Supplementary Figure S1 — Pairwise comparison of 13 quality control (QC) samples transformed by log10. Each point in the scatter plot represents a metabolite. All the points are distributed in a tight straight line, and the corresponding correlation coefficient is >0.99, indicating that the data is highly consistent in the two QC samples. On the diagonal is the data distribution diagram of a single sample. [file Data_Sheet_1.zip › Figure 3.TIFF]
