# Supplementary material for: Metabolomics-Based Frailty Biomarkers in Older Chinese Adults
Source: Front Med (Lausanne). 2022 Jan 26;8:830723. doi: 10.3389/fmed.2021.830723 (PMC8825494; doi:10.3389/fmed.2021.830723)
Supplement: Supplementary Table S1 — Pearson correlation of metabolites associated with grip strength. [file Table_1.docx]

Supplemental materials for

**Metabolomics-based frailty biomarkers in Chinese older adults**

**Table S1. Pearson correlation of metabolites associated with grip strength.**

| **Metabolite** | **HMDB** | **Class** | **Pearson r** | ***p* value** |
| --- | --- | --- | --- | --- |
| Phytosphingosine | HMDB0004610 | Amines | 0.3922 | 0.0018 |
| L-Lysine | HMDB0000182 | Amino acids | 0.2838 | 0.0267 |
| N6-Acetyl-L-lysine | HMDB0000206 | Amino acids | -0.3035 | 0.0174 |
| 5-Hydroxy-L-tryptophan | HMDB0000472 | Amino acids | -0.2669 | 0.0376 |
| Leucyl-Serine | HMDB0028938 | Amino acids | -0.2536 | 0.0486 |
| Thr Ile Tyr Phe | METLIN 234663 | Amino acids | -0.3876 | 0.0020 |
| Thr Leu Tyr Phe | METLIN 235463 | Amino acids | -0.2874 | 0.0247 |
| L-2-Aminoadipic acid | PubChem 92136 | Amino acids | 0.2595 | 0.0435 |
| 3-Methylphenylacetic acid | HMDB0002222 | Benzene | -0.3019 | 0.0180 |
| p-Toluenesulfonic acid | HMDB0059933 | Benzene | -0.4023 | 0.0013 |
| Glycocholic acid | HMDB0000138 | Bile acids | 0.2659 | 0.0384 |
| Deoxycholic acid glycine conjugate | HMDB0000631 | Bile acids | 0.3931 | 0.0017 |
| Glycoursodeoxycholic acid | HMDB0000708 | Bile acids | 0.3022 | 0.0179 |
| Taurodeoxycholic acid | HMDB0000896 | Bile acids | 0.4370 | 0.0004 |
| Gluconic acid | HMDB0000625 | Carbohydrates | -0.3984 | 0.0015 |
| 1-(beta-D-Ribofuranosyl)-1,4-dihydronicotinamide | HMDB0011648 | Carbohydrates | -0.3092 | 0.0153 |
| Fumaric acid | HMDB0000134 | Carboxylic acids | -0.2844 | 0.0263 |
| 3,3'-Thiobispropanoic acid | HMDB0031162 | Carboxylic acids | -0.2887 | 0.0241 |
| LysoPC(14:0) | HMDB0010379 | Glycerophosphocholines | -0.3600 | 0.0044 |
| sn2 LysoPC(14:0) | — | Glycerophosphocholines | -0.3520 | 0.0054 |
| 1-Stearoylglycerophosphoglycerol | HMDB0061697 | Glycerophosphoglycerols | 0.3061 | 0.0164 |
| Tryptophan 2-C-mannoside | HMDB0240296 | Indolyl carboxylic acids | -0.3528 | 0.0053 |
| Pseudouridine | HMDB0000767 | Nucleosides | -0.2973 | 0.0200 |
| 1-Methyladenosine | HMDB0003331 | Purine nucleosides | -0.2983 | 0.0195 |
| Succinyladenosine | HMDB0000912 | Purine nucleosides | -0.2582 | 0.0445 |
| 1-Methylguanine | HMDB0003282 | Purines | -0.2874 | 0.0247 |
| Theophylline | HMDB0001889 | Purines | 0.2654 | 0.0387 |
| 3-Pyridylacetic acid | HMDB0001538 | Pyridines | -0.2844 | 0.0263 |
| Ribothymidine | HMDB0000884 | Pyrimidine nucleosides | 0.2587 | 0.0441 |
| Fusicoccin H | METLIN 67422 | Steroidal glycosides | 0.3267 | 0.0102 |
| Cyclamic acid | HMDB0031340 | Sulfamic acid | -0.2841 | 0.0265 |
| Biotin | HMDB0000030 | Vitamin | -0.3108 | 0.0148 |

**Table S2. Pearson correlation of metabolites associated with gait speed.**

| **Metabolite** | **HMDB** | **Class** | **Pearson r** | ***p* value** |
| --- | --- | --- | --- | --- |
| L-Palmitoylcarnitine | HMDB0000222 | Acyl carnitines | -0.3878 | 0.0013 |
| Stearoylcarnitine | HMDB0000848 | Acyl carnitines | -0.2945 | 0.0164 |
| Dodecanoylcarnitine | HMDB0002250 | Acyl carnitines | -0.2563 | 0.0378 |
| Oleoylcarnitine | HMDB0005065 | Acyl carnitines | -0.2777 | 0.0240 |
| Tetradecanoylcarnitine | HMDB0005066 | Acyl carnitines | -0.4121 | 0.0006 |
| trans-2-Tetradecenoylcarnitine | HMDB0013329 | Acyl carnitines | -0.2560 | 0.0380 |
| Isovalerylcarnitine | HMDB0000688 | Acyl carnitines | -0.2440 | 0.0484 |
| cis-5-Tetradecenoylcarnitine | HMDB0002014 | Acyl carnitines | -0.2688 | 0.0291 |
| trans-Hexadec-2-enoyl carnitine | HMDB0006317 | Acyl carnitines | -0.3803 | 0.0016 |
| 9,12-Hexadecadienoylcarnitine | HMDB0013334 | Acyl carnitines | -0.3003 | 0.0143 |
| Isobutyrylcarnitine | HMDB0000736 | Acyl carnitines | -0.2591 | 0.0357 |
| L-Alanine | HMDB0000161 | Amino acids | -0.2590 | 0.0357 |
| N6-Acetyl-L-lysine | HMDB0000206 | Amino acids | -0.3287 | 0.0070 |
| 5-Hydroxy-L-tryptophan | HMDB0000472 | Amino acids | -0.2628 | 0.0331 |
| 4-Hydroxyproline | HMDB0000725 | Amino acids | -0.2785 | 0.0235 |
| N-Acetyl-L-alanine | HMDB0000766 | Amino acids | -0.3142 | 0.0102 |
| 5-methoxy-L-tryptophan | HMDB0002339 | Amino acids | 0.2450 | 0.0474 |
| Gamma-Glutamyltyrosine | HMDB0011741 | Amino acids | -0.2701 | 0.0283 |
| N-Acetylvaline | HMDB0011757 | Amino acids | -0.2657 | 0.0311 |
| Gamma-Glutamyl Glutamine | HMDB0028833 | Amino acids | -0.2603 | 0.0348 |
| Phenylalanyltryptophan | HMDB0029006 | Amino acids | -0.2648 | 0.0316 |
| Tyrosyl-Alanine | HMDB0029098 | Amino acids | 0.2464 | 0.0461 |
| N-gamma-L-Glutamyl-L-methionine | HMDB0034367 | Amino acids | -0.2623 | 0.0334 |
| N-Formyl-L-methionine | HMDB0001015 | Amino acids | -0.3234 | 0.0081 |
| Alpha-N-Phenylacetyl-L-glutamine | HMDB0006344 | Amino acids | -0.3125 | 0.0106 |
| Pro Tyr Tyr Val | METLIN 207476 | Amino acids | -0.2981 | 0.0150 |
| Thr Ile Phe Tyr | METLIN 234378 | Amino acids | -0.2952 | 0.0161 |
| Thr Ile Tyr Phe | METLIN 234663 | Amino acids | -0.3734 | 0.0020 |
| Thr Leu Phe Tyr | METLIN 235178 | Amino acids | -0.2887 | 0.0187 |
| Thr Leu Tyr Phe | METLIN 235463 | Amino acids | -0.3045 | 0.0129 |
| Thr Tyr Phe Ile | METLIN 239166 | Amino acids | -0.3337 | 0.0062 |
| N-Acetyl-L-leucyl-L-proline | METLIN 837905 | Amino acids | -0.2621 | 0.0335 |
| 3-Methylphenylacetic acid | HMDB0002222 | Benzene | -0.3022 | 0.0137 |
| Phenacemide | HMDB0015253 | Benzene | -0.3029 | 0.0134 |
| 4-Hydroxyhippuric acid | HMDB0013678 | Benzoic acids | -0.2648 | 0.0316 |
| D-Glucuronic acid | HMDB0000127 | Carbohydrates | -0.4415 | 0.0002 |
| Glyceric acid | HMDB0000139 | Carbohydrates | 0.3636 | 0.0027 |
| Gluconic acid | HMDB0000625 | Carbohydrates | -0.2760 | 0.0249 |
| N-Acetylgalactosamine 6-sulfate | HMDB0000841 | Carbohydrates | -0.2629 | 0.0330 |
| L-Threonic Acid | HMDB0000943 | Carbohydrates | 0.2784 | 0.0236 |
| Arabinonic acid | HMDB0000539 | Carbohydrates | -0.2434 | 0.0489 |
| Muramic acid | HMDB0003254 | Carbohydrates | -0.2595 | 0.0354 |
| 1-(beta-D-Ribofuranosyl)-1,4-dihydronicotinamide | HMDB0011648 | Carbohydrates | -0.3879 | 0.0013 |
| cis-Aconitic acid | HMDB0000072 | Carboxylic acids | -0.4461 | 0.0002 |
| Citric acid | HMDB0000094 | Carboxylic acids | -0.3275 | 0.0073 |
| Fumaric acid | HMDB0000134 | Carboxylic acids | -0.2706 | 0.0280 |
| Isocitric acid | HMDB0000193 | Carboxylic acids | -0.3981 | 0.0009 |
| 3,3'-Thiobispropanoic acid | HMDB0031162 | Carboxylic acids | -0.2579 | 0.0366 |
| Itaconic acid | HMDB0002092 | Fatty acids | -0.3864 | 0.0014 |
| 9,10-DHOME | HMDB0004704 | Fatty acids | -0.2789 | 0.0233 |
| 3-Oxododecanoic acid | HMDB0010727 | Fatty acids | -0.2446 | 0.0478 |
| 3-Hydroxytetradecanedioic acid | HMDB0000394 | Fatty acids | -0.3830 | 0.0015 |
| 2-methyl-tridecanedioic acid | LMFA01170015 | Fatty acids | -0.2484 | 0.0443 |
| 13(S)-HpOTrE | METLIN 36052 | Fatty acids | -0.2598 | 0.0351 |
| 13-HODE | HMDB0004667 | Fatty Acyls | -0.3522 | 0.0037 |
| LysoPC(14:0) | HMDB0010379 | Glycerophosphocholines | -0.2937 | 0.0167 |
| LysoPC(16:1) | HMDB0010383 | Glycerophosphocholines | -0.2730 | 0.0266 |
| sn2 LysoPC(14:0) | — | Glycerophosphocholines | -0.2942 | 0.0165 |
| LysoPE(18:2) | HMDB0011507 | Glycerophosphoethanolamines | -0.2698 | 0.0285 |
| sn2 LysoPE(18:2) | — | Glycerophosphoethanolamines | -0.2713 | 0.0276 |
| L-Malic acid | HMDB0000156 | Hydroxy acids | -0.3143 | 0.0102 |
| 3-Hydroxydodecanedioic acid | HMDB0000413 | Hydroxy acids | -0.3136 | 0.0103 |
| 3-Hydroxycapric acid | HMDB0002203 | Hydroxy acids | -0.2492 | 0.0436 |
| 2,4-Dihydroxybutanoic acid | HMDB0000360 | Hydroxy acids | -0.3718 | 0.0021 |
| 5-Hydroxyindoleacetic acid | HMDB0000763 | Indoles | -0.2950 | 0.0162 |
| Tryptophan 2-C-mannoside | HMDB0240296 | Indolyl carboxylic acids | -0.3405 | 0.0051 |
| 2-Ketobutyric acid | HMDB0000005 | Keto acids | -0.2466 | 0.0460 |
| Pyruvic acid | HMDB0000243 | Keto acids | -0.3007 | 0.0142 |
| Pseudouridine | HMDB0000767 | Nucleosides | -0.3932 | 0.0011 |
| p-Cresol sulfate | HMDB0011635 | Organic sulfuric acids | -0.2489 | 0.0439 |
| L-Kynurenine | HMDB0000684 | Organooxygen | -0.2780 | 0.0238 |
| 1-Methyladenosine | HMDB0003331 | Purine nucleosides | -0.2471 | 0.0455 |
| Succinyladenosine | HMDB0000912 | Purine nucleosides | -0.2862 | 0.0198 |
| Hypoxanthine | HMDB0000157 | Purines | 0.2678 | 0.0297 |
| 1-Methylguanine | HMDB0003282 | Purines | -0.4512 | 0.0001 |
| 3-Pyridylacetic acid | HMDB0001538 | Pyridines | -0.3920 | 0.0011 |
| 4-Methylpyrrolo[1,2-a]pyrazine | HMDB0033173 | Pyrrolopyrazines | 0.2780 | 0.0238 |
| Acetylcholine | HMDB0000895 | Quaternary ammonium salts | -0.2841 | 0.0208 |
| Etiocholanolone glucuronide | HMDB0004484 | Steroidal glycosides | -0.2855 | 0.0201 |
| Pregnanediol-3-glucuronide | HMDB0010318 | Steroidal glycosides | -0.3705 | 0.0022 |
| Fusicoccin H | METLIN 67422 | Steroidal glycosides | 0.3408 | 0.0051 |
| (25S)-11alpha,20,26-trihydroxyecdysone | METLIN 57610 | Steroids | -0.2811 | 0.0222 |
| Biotin | HMDB0000030 | Vitamin | -0.2680 | 0.0296 |
